# Effectiveness and usability of an e-health system on depression among patients with somatic disorders

**DOI:** 10.1192/j.eurpsy.2024.78

**Published:** 2024-08-27

**Authors:** T. Vitcheva, N.G. Petros, G. Hadlaczky, V. Carli

**Affiliations:** ^1^Karolinska Institutet, Stockholm, Sweden

## Abstract

**Introduction:**

An increase in the prevalence of depressive symptoms can be seen in patients with severe somatic conditions, with a reduction in quality of life, an increase in sleep disturbances and an increased risk of suicide as some of the most serious consequences. However, few evidence-based interventions have been developed with the aim of reducing this comorbidity. The NEVERMIND system aims to address this issue by collecting psychometric and biomedical data via a smart shirt and a mobile app, which are used to predict patients’ depressive symptoms. Patients are then directed to personalised lifestyle behavioural advice, mindfulness-based therapy, and cognitive behavioural therapy.

**Objectives:**

The primary objective was to evaluate the effectiveness of the NEVERMIND system in reducing depressive symptoms in patients with somatic conditions compared to treatment as usual. Secondary objectives included the system’s effectiveness in preventing depressive symptoms, sustaining the effects at 24 weeks post-baseline, and reducing suicide ideation. Besides these, the usability, acceptability, and satisfaction of the system were examined in patients with breast or prostate cancer.

**Methods:**

For this pragmatic randomised controlled trial, 425 patients diagnosed with myocardial infarction, breast or prostate cancer, kidney failure, or lower limb amputation were recruited from hospitals in Turin, Pisa and Lisbon. Data collection occurred at baseline, 12 weeks, and 24 weeks, with the primary outcome being depressive symptoms at week 12, measured by the Beck Depression Inventory II. Regarding the usability, acceptability and patient satisfaction, data from 288 patients was used.

**Results:**

The intervention group included 213 and the control group 212 patients, with the sample’s mean age being 59.41 (SD=10.70). Patients who used the system reported having statistically significant lower depressive symptoms at 12 weeks (mean difference=-3.05, p=0.004; 95%CI -5.12 to -0.99) compared to controls, with a clinically relevant effect size (Cohen’s d=0.41). Furthermore, significant reductions were found for suicide ideation (mean difference=-0.61, p=0.020; 95%CI -1.13 to -0.10) and incidence of depressive symptoms at week 12 (OR=0.43, p=0.019; 95%CI 0.22 to 0.87). The decrease in depressive symptoms was sustained at week 24 (mean difference=-1.34, p=0.015; 95%CI -2.41 to -0.26). The system was found to have good usability, with women rating the system more favourably than men and valuing its emotional support, while men used the system more frequently than women and valued the self-awareness that the system encouraged.

**Conclusions:**

The NEVERMIND system was shown to be superior to standard care in reducing and preventing depressive symptoms among the studied sample. A new project will be launched in the near future to continue the examination of the system’s effectiveness.

**Disclosure of Interest:**

None Declared

